# Seasonal fluctuations of CGM metrics in individuals with type 1 diabetes using an intermittently scanned CGM device or sensor augmented pump

**DOI:** 10.1007/s12020-024-03971-5

**Published:** 2024-07-25

**Authors:** Yuka Oi-Yo, Shin Urai, Akane Yamamoto, Tomofumi Takayoshi, Masaaki Yamamoto, Yushi Hirota, Wataru Ogawa

**Affiliations:** Division of Diabetes and Endocrinology, Department of Internal Medicine, Kobe University Graduate School of Medicine, Kobe, Japan

**Keywords:** Type 1 diabetes mellitus, Continuous glucose monitoring (CGM), Sensor augmented pump (SAP), Seasonal fluctuation, Time in range (TIR)

## Abstract

**Objective:**

To elucidate the fluctuations in glucose levels measured using CGM-metrics during the four distinct seasons of the year in individuals with type 1 diabetes mellitus (T1DM) using an intermittently scanned CGM (isCGM) device or sensor augmented pump (SAP).

**Research design and methods:**

This retrospective, single-center study enrolled 93 individuals with T1DM who were equipped with an isCGM device or SAP at Kobe University Hospital. The subjects had a median age of 47.0 years [interquartile range, 37.0–62.0 years], 25 individuals (26.9%) were male, median body mass index was 22.0 kg/m^2^ [20.8–23.8 kg/m^2^], and median hemoglobin A_1c_ level was 7.4% [6.9–8.0%]. CGM data were reviewed from January to December 2019, and the mean sensor glucose (SG) value, time above range (TAR), time in range (TIR), time below range (TBR), and standard deviation (SD) of SG were calculated for each season (spring, March–May; summer, June–August; autumn, September–November; winter, December–February).

**Results:**

Seasonal fluctuations were detected for mean SG, TAR, TIR, and SD, with TIR being lower and mean SG, TAR, and SD being higher in cold seasons (spring or winter) than in warm seasons (summer or autumn).

**Conclusion:**

Seasonal fluctuations in CGM metrics should be taken into account in future studies performed to evaluate the favorable impact of CGM on glycemic management in individuals with T1DM.

## Introduction

Glycosylated hemoglobin (HbA_1c_) reflects the average blood glucose level over the preceding several months and has served as a surrogate marker for glycemic control [1, 2]. However, there are limitations to evaluation of certain aspects of blood glucose control with this parameter. It thus does not provide information on rapid fluctuations in blood glucose concentration, the occurrence of hypoglycemia or hyperglycemia, or the extent and frequency of intraday blood glucose changes [2]. In addition, measurements of HbA_1c_ levels may be falsely low or high under specific circumstances. Continuous glucose monitoring (CGM) provides insight into changes in blood glucose levels that cannot be determined on the basis of HbA_1c_ measurement. CGM devices have advanced markedly in recent years, with their use having made possible the monitoring of blood glucose levels more accurately.

Given that many studies have shown the utility of CGM, CGM-based metrics of glycemic control have been proposed [2]. Key CGM metrics include the percentage of readings and time per day within the target glucose range (TIR), time below the target glucose range (TBR), and time above the target glucose range (TAR). Long-term studies are needed to determine whether the use of these metrics is related to clinical outcomes such as diabetic complications and mortality. A relation between TIR and HbA_1c_ level was recently demonstrated [3, 4], as was a relation between TIR and diabetic complications [5, 6], suggesting that adoption of CGM metrics may help to extend healthy life expectancy in individuals with diabetes mellitus. There are two types of CGM—intermittently scanned CGM (isCGM) and real-time CGM (rtCGM)—with the use of each type having been shown to improve glycemic management and to reduce hypoglycemic time as well as diabetic complications and mortality compared with self-monitoring of blood glucose (SMBG) alone [7–10].

The establishment of targets treatment goals for each disease state is important for determination of the most effective therapy. Treatment options for diabetes vary depending on the combination of CGM and insulin administration method (such as multiple daily injections [MDI] or continuous subcutaneous insulin infusion [CSII]), and it is important to consider the best treatment for each individual. Investigation of clinical differences in CGM metrics between isCGM and a sensor augmented pump (SAP) would be meaningful for provision of appropriate device-based therapies for individuals with diabetes mellitus.

Individuals who show seasonal fluctuations in HbA_1c_ levels are often encountered in the clinical setting, and the need for glycemic control that takes such fluctuations into account has been widely noted, regardless of the type of diabetes mellitus, patient ethnicity, or geographic region [11–15]. However, whether CGM metrics, including diurnal variation in blood glucose, also show similar seasonal fluctuations has remained unknown.

In Japan, SAP, isCGM, and standalone rtCGM devices have been covered by insurance since 2014, 2017, and 2018, respectively. As of 2019, most CGM devices in use were isCGM or SAP systems. The aim of this study is to elucidate the fluctuations in glucose levels measured using CGM-metrics during the four distinct seasons of the year in individuals with type 1 diabetes mellitus (T1DM) fitted with an isCGM device or SAP.

## Materials and methods

### Subjects, study design, and data collection

This retrospective, single-center study was conducted at Kobe University Hospital. Of the 182 consecutive adults with T1DM referred to our department at this tertiary medical institution in 2019, 89 individuals were excluded on the basis of the following criteria: (1) missing CGM data for >4 consecutive months; (2) sensor wearing for <70% of the time on average per year; (3) use of a standalone rtCGM device, given that such devices were highly uncommon in Japan in 2019; (4) a change in treatment method during the target period, including a switch to a new type of insulin pump (such as from a Minimed 620 G to a Minimed 640 G pump [Medtronic, Northridge, CA, USA]); (5) dialysis, pregnancy, or steroid use. The criteria for study inclusion did not extend to encompass a range of HbA_1c_ levels. The remaining 93 individuals were enrolled in the study. The CGM device worn by the study subjects was FreeStyle Libre (Abbott, Witney, UK) for isCGM *(n* = 50) or Enlite Sensor (Medtronic) for SAP *(n* = 43). Each isCGM device was first generation, before the algorithm modification.

We collected data from January to December 2019 in order to exclude the influence of the reduced frequency of physical activity due to the COVID-19 pandemic that was apparent in Japan beginning in 2020 [16, 17]. We thus investigated potential seasonal fluctuations in CGM metrics for individuals with T1DM who were equipped with an isCGM device or SAP and attended our hospital. The data were analyzed for the study population as a whole as well as for the isCGM and SAP groups separately.

The study was approved by the Research Ethics Committee of Kobe University Hospital (approval no. B220218). Individuals had the option to opt out of the study after they were provided with information explaining its purpose and the data to be collected.

### CGM metrics and HbA_1c_ level

The Japan Meteorological Agency defines the seasons as follows: March to May, spring; June to August, summer; September to November, autumn; and December to February, winter [18]. We collected CGM data for each individual and calculated the mean sensor glucose (SG) value, TAR ( > 180 mg/dL [10 mmol/L]), TIR (70–180 mg/dL [3.9–10 mmol/L]), TBR ( < 70 mg/dL [3.9 mmol/L]), and standard deviation (SD) of SG during each season. HbA_1c_ was measured by high-performance liquid chromatography with an HA8181 system (Arkray, Kyoto, Japan).

Nine individuals were excluded from the analysis of HbA_1c_ levels if there were consecutive missing data points.

### Other measurements

We investigated the following clinical characteristics as obtained from medical records: sex, age, body mass index (BMI), disease duration, the presence of complications (neuropathy, retinopathy, nephropathy, or history of cardiovascular disease), and insulin administration method (MDI or CSII). Evaluation of diabetic neuropathy was based on symptoms, quantitative sensory testing (vibration and monofilament tests), and quantitative motor testing (patellar and ankle reflexes) [19]. Diabetic retinopathy was categorized as nonproliferative, preproliferative, or proliferative [19]. Diabetic nephropathy was defined by measurement of albumin levels in 24-h urine samples (normal value: <30 mg/day); microalbuminuria and macroalbuminuria were diagnosed if the albumin excretion rate was 30 to 300 or >300 mg/day, respectively [20]. Diabetic nephropathy was confirmed by the absence of signs and symptoms due to other primary causes of kidney disease.

### Statistical analysis

Statistical analysis was performed with GraphPad Prism 10 (GraphPad, San Diego, CA, USA) and EZR software [21]. Continuous variables were analyzed with statistical graphics, and the Shapiro-Wilk normality test was performed to confirm a normal distribution. Differences in data between two groups were assessed with the Mann-Whitney *U* test. One-way repeated measures analysis of variance (ANOVA) with post hoc Bonferroni’s correction was adopted to evaluate differences among seasons. The chi-square test or Fisher’s exact test was applied to the analysis of categorical data. Results are presented as median [interquartile range]. A *P* value of <0.05 was considered statistically significant.

## Results

### Clinical characteristics

The baseline clinical characteristics of the study subjects are shown in Table 1. Median age was 47.0 [37.0–62.0] years, 25 individuals (26.9%) were male and 56 (60.2%) used CSII, median BMI was 22.0 [20.8–23.8] kg/m^2^, median HbA_1c_ level was 7.4% [6.9–8.0%] or 57.4 [51.9–63.9] mmol/mol, and the median of the mean SG level on CGM was 159.3 [139.8–180.8] mg/dL or 8.8 [7.8–10.0] mmol/L. Compared with the SAP group, the isCGM group had a lower BMI (21.5 [20.5–22.8] *vs.* 22.8 [21.5–24.3] kg/m^2^) and higher HbA_1c_ level (7.8% [7.1–8.5%] *vs.* 7.1% [6.9–7.7%], or 61.7 [54.1–69.4] *vs.* 54.1 [51.9–60.6] mmol/mol). However, there was no significant difference in sex distribution (26.0% *vs.* 27.9% male), age (52.0 [39.0–64.8] *vs.* 44.0 [36.0–56.0] years), or disease duration (12.5 [7.0–18.0] *vs.* 14.0 [6.0–18.5] years) between the isCGM and SAP groups, respectively.

**Table 1:** Baseline clinical characteristics of the study subjects with type 1diabetes mellitus fitted with an isCGM device or SAP

Characteristic	Total (*n* = 93)	isCGM (*n* = 50)	SAP (*n* = 43)	*P* value
Age (years)	47.0 [37.0–62.0]	52.0 [39.0–64.8]	44.0 [36.0–56.0]	0.08
Male, *n* (%)	25 (26.9)	13 (26.0)	12 (27.9)	0.99
Body mass index (kg/m^2^)	22.0 [20.8–23.8]	21.5 [20.5–22.8]	22.8 [21.5–24.3]	0.04
Duration of diabetes (years)	13.0 [6.0–18.0]	12.5 [7.0–18.0]	14.0 [6.0–18.5]	0.90
CSII, *n* (%)	56 (60.2)	13 (26.0)	43 (100)	<0.01
HbA_1c_ (%)	7.4 [6.9–8.0]	7.8 [7.1–8.5]	7.1 [6.9–7.7]	<0.01
HbA_1c_ (mmol/mol)	57.4 [51.9–63.9]	61.7 [54.1–69.4]	54.1 [51.9–60.6]	<0.01
CGM metrics				
Mean SG (mg/dL)	159.3 [139.8–180.8]	177.4 [148.4–191.5]	148.8 [134.9–176.3]	<0.01
Mean SG (mmol/L)	8.8 [7.8–10.0]	9.8 [8.2–10.6]	8.3 [7.5–9.8]	<0.01
TAR (%)	32.6 [20.9–46.3]	43.3 [26.3–54.3]	25.2 [17.6–42.1]	<0.01
TIR (%)	62.4 [50.2–72.5]	51.6 [44.2–60.6]	70.5 [55.9–77.7]	<0.01
TBR (%)	3.4 [1.3–7.0]	4.8 [1.3–9.6]	2.3 [0.8–5.5]	0.04
SD (mg/dL)	59.2 [50.0–69.8]	69.6 [60.0–75.2]	53.6 [46.5–61.7]	<0.01
SD (mmol/L)	3.3 [2.8–3.9]	3.9 [3.3–4.2]	3.0 [2.6–3.4]	<0.01
Complications				
Retinopathy, *n* (%)	20 (21.5)	13 (26.0)	7 (16.3)	0.32
Renal disease, *n* (%)	13 (14.0)	9 (18.0)	4 (9.3)	0.48
Neuropathy, *n* (%)	23 (24.7)	9 (18.0)	14 (32.6)	0.15
CVD, *n* (%)	3 (3.2)	3 (6.0)	0 (0.0)	0.25

Data are presented as median [interquartile range] or as *n* (%). The *P* values for comparisons between the isCGM and SAP groups were determined with the Mann-Whitney *U* test for continuous variables and with the chi-square test or Fisher’s exact test for categorical variables. Nine individuals (5 in isCGM and 4 in SAP group) were excluded from the analysis of HbA_1c_ levels because there were consecutive missing data points

*CGM* continuous glucose monitoring, *isCGM* intermittently scanned CGM, *SAP* sensor augmented pump, *CSII* continuous subcutaneous insulin infusion, *HbA*_*1c*_ hemoglobin A_1c_, *SG* sensor glucose, *TAR* time above range, *TIR* time in range, *TBR* time below range, *SD* standard deviation, *CVD* cardiovascular disease

### Seasonal fluctuations in HbA_1c_ levels and CGM metrics in individuals with T1DM

**Fig. 1:**
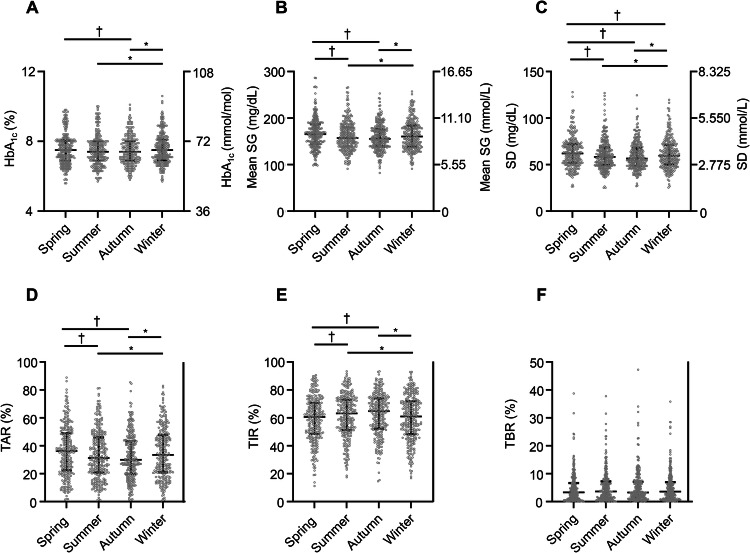
Seasonal fluctuations in HbA_1c_ level and CGM metrics for the overall study population. Seasonal fluctuations in HbA_1c_ level (**A**), mean SG (**B**), SD (**C**), TAR (**D**), TIR (**E**), and TBR (**F**) are shown. *P < 0.05 for comparisons with the value for winter; †*P* < 0.05 for comparisons with the value for spring (One-way repeated measures analysis of variance (ANOVA) with Bonferroni’s correction). *HbA*_*1c*_ hemoglobin A_1c_, *CGM* continuous glucose monitoring, *SG* sensor glucose, *SD* standard deviation, *TAR* time above range, *TIR* time in range, *TBR* time below range

We investigated whether CGM metrics showed seasonal fluctuations for the study population overall. None of the individuals included in the study experienced a severe acute illness during the observation period. TIR was higher and mean SG and TAR were lower in summer or autumn than in spring or winter (TIR: spring *vs*. summer, *P* < 0.01; spring *vs*. autumn, *P* < 0.01; summer *vs*. winter, *P* = 0.02; autumn *vs*. winter, *P* < 0.01) (mean SG: spring *vs*. summer, *P* < 0.01; spring *vs*. autumn, *P* < 0.01; summer *vs*. winter, *P* = 0.03; autumn *vs*.winter, *P* < 0.01) (TAR: spring *vs*. summer, *P* < 0.01; spring *vs*. autumn, *P* < 0.01; summer *vs*. winter, *P* < 0.01, autumn *vs*. winter, *P* < 0.01). SD was lower in winter than in spring, in addition to showing a similar trend to these parameters (spring *vs*. summer, *P* < 0.01; spring *vs*. autumn, *P* < 0.01; spring *vs*. winter, *P* = 0.02; summer *vs*. winter, *P* = 0.03; autumn *vs*. winter, *P* < 0.01) (Fig. 1, Table 2). However, there was no significant seasonal fluctuation apparent for TBR. HbA_1c_ levels were lower in summer or autumn than in winter, and lower in autumn than in spring (summer *vs*. winter, *P* < 0.01; autumn *vs*. winter, *P* < 0.01; spring *vs*. autumn, *P* = 0.04), but, unlike the CGM metrics, they did not differ between summer versus spring.

**Table 2:** Seasonal differences in CGM metrics and HbA_1c_ levels for the overall study population as well as for the isCGM and SAP groups separately

*Parameter*	*Spring*	*Summer*	*Autumn*	*Winter*
Total				
HbA_1c_ (%)	7.5 [6.9–8.0]	7.4 [6.9–8.0]*	7.4 [6.9–8.0]*†	7.5 [7.0–8.1]
HbA_1c_ (mmol/mol)	58.5 [51.9–63.9]	57.4 [51.9–63.9]*	57.4 [51.9–63.9]*†	58.5 [51.9–63.9]
Mean SG (mg/dL)	165.3 [144.1–186.1]	157.1 [139.1–180.0]*†	155.4 [139.1–175.6]*†	160.3 [138.9–183.7]
Mean SG (mmol/L)	9.2 [8.0–10.3]	8.7 [7.7–10.0]*†	8.6 [7.7–9.7]*†	8.9 [7.7–10.2]
SD (mg/dL)	61.9 [51.9–72.1]	58.2 [49.4–68.8]*†	56.6 [48.7–67.5]*†	59.3 [50.2–71.0]†
SD (mmol/L)	3.4 [2.9–4.0]	3.2 [2.7–3.8]*†	3.1 [2.7–3.7]*†	3.3 [2.8–3.9]†
TAR (%)	36.3 [22.7–48.9]	31.2 [20.8–45.8]*†	30.0 [19.9–43.3]*†	33.4 [21.1–47.7]
TIR (%)	60.6 [48.5–70.7]	63.2 [51.2–72.9]*†	64.8 [52.6–73.9]*†	61.0 [48.3–71.9]
TBR (%)	3.3 [1.1–6.6]	3.6 [1.3–7.2]	3.2 [1.5–7.1]	3.6 [1.1–7.0]
isCGM group				
Mean SG (mg/dL)	169.9 [155.7–190.6]	164.5 [148.7–187.6] †	161.8 [146.5–180.9]*†‡	170.1 [148.5–190.2]
Mean SG (mmol/L)	9.4 [8.6–10.6]	9.1 [8.3–10.4] †	9.0 [8.1–10.0]*†‡	9.4 [8.2–10.6]
SD (mg/dL)	68.6 [59.3–76.3]	64.0 [55.2–75.8] †	62.6 [54.4–72.0]*†‡	65.2 [56.5–75.5]
SD (mmol/L)	3.8 [3.3–4.2]	3.6 [3.1–4.2] †	3.5 [3.0–4.0]*†‡	3.6 [3.1–4.2]
TAR (%)	40.2 [31.2–52.8]	37.4 [26.2–49.9] †	36.4 [26.0–47.1]*†‡	40.3 [26.7–53.6]
TIR (%)	53.0 [43.5–62.6]	56.8 [45.1–65.1] †	58.3 [46.1–66.4]*†‡	54.9 [44.1–63.6]
TBR (%)	3.9 [1.3–8.0]	4.5 [1.4–8.7] †	4.9 [1.8–8.8] †	4.7 [1.3–8.9]
SAP group				
Mean SG (mg/dL)	149.8 [132.8–171.4]	147.2 [131.3–170.6]*†	148.9 [136.7–170.4]	147.3 [134.3–176.0]
Mean SG (mmol/L)	8.3 [7.4–9.5]	8.2 [7.3–9.5]*†	8.3 [7.6–9.5]	8.2 [7.5–9.8]
SD (mg/dL)	53.2 [47.2–63.1]	51.3 [46.2–60.0]†	52.2 [46.2–58.4]	53.3 [46.2–60.6]
SD (mmol/L)	3.0 [2.6–3.5]	2.8 [2.6–3.3]†	2.9 [2.6–3.2]	3.0 [2.6–3.4]
TAR (%)	26.2 [16.3–39.8]	23.7 [14.5–39.7]*†	26.3 [15.9–37.6]	24.4 [16.0–42.6]
TIR (%)	69.5 [58.2–76.2]	72.3 [58.5–78.0]†	71.4 [61.9–77.6] †	70.6 [55.5–78.7]
TBR (%)	2.9 [0.9–5.7]	3.0 [1.2–6.4]	2.7 [1.2–4.8]	2.5 [0.9–5.7]

Data are presented as median [interquartile range]. *P < 0.05 *vs.* the corresponding value for winter; †*P* < 0.05 *vs.* the corresponding value for spring; ‡*P* < 0.05 *vs.* the corresponding value for summer (One-way repeated measures analysis of variance (ANOVA) with Bonferroni’s correction)

*CGM* continuous glucose monitoring, *HbA*_*1c*_ hemoglobin A_1c_, *SG* sensor glucose, *SD* standard deviation, *TAR* time above range, *TIR* time in range, *TBR* time below range, *isCGM* intermittently scanned CGM, *SAP* sensor augmented pump

### Seasonal fluctuations in CGM metrics for the isCGM and SAP groups

**Fig. 2:**
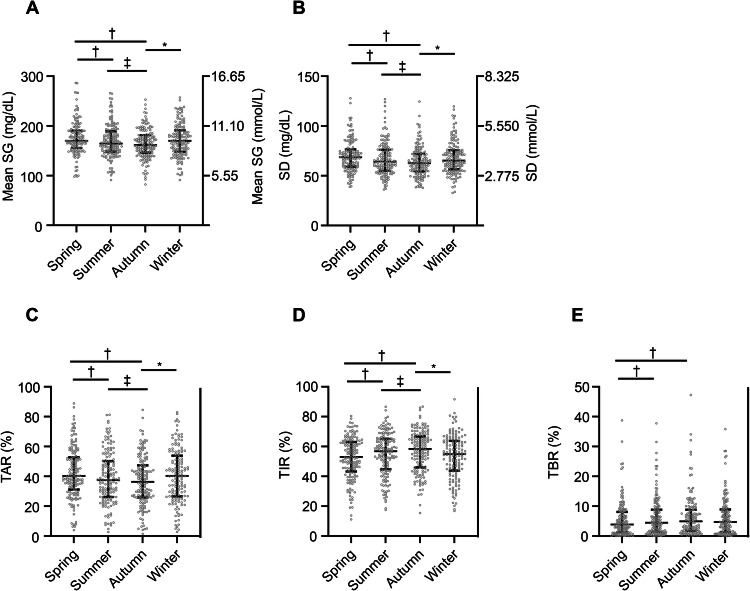
Seasonal fluctuations in CGM metrics for the isCGM group of subjects. Seasonal fluctuations in mean SG (**A**), SD (**B**), TAR (**C**), TIR (**D**), and TBR (**E**) are shown. *P < 0.05 for comparisons with the value for winter; †*P* < 0.05 for comparisons with the value for spring; ‡*P* < 0.05 for comparisons with the value for summer (One-way repeated measures analysis of variance (ANOVA) with Bonferroni’s correction). *CGM* continuous glucose monitoring, *isCGM* intermittently scanned CGM, *SG* sensor glucose, *SD* standard deviation, *TAR* time above range, *TIR* time in range, *TBR* time below range

We also investigated seasonal fluctuations in CGM metrics for the isCGM and SAP groups separately. In the isCGM group, seasonal fluctuations were observed for mean SG, SD, TAR, TIR, and TBR. Mean SG, SD and TAR were lower in autumn than in spring, summer, or winter and in summer than in spring (mean SG: spring *vs*. summer, *P* < 0.01; spring *vs*. autumn, *P* < 0.01; summer *vs*. autumn, *P* < 0.01; autumn *vs*. winter, *P* < 0.01) (SD: spring *vs*. summer, *P* < 0.01; spring *vs*. autumn, *P* < 0.01; summer *vs*. autumn, *P* < 0.01; autumn *vs*. winter, *P* < 0.01) (TAR: spring *vs*. summer, *P* < 0.01; spring *vs*. autumn, *P* < 0.01; summer *vs*. autumn, *P* = 0.01; autumn *vs*. winter, *P* < 0.01) (Fig. 2, Table 2). Similarly, TIR was higher in autumn than in spring, summer, or winter and in summer than in spring (spring *vs*. summer, *P* < 0.01; spring *vs*. autumn, *P* < 0.01; summer *vs*. autumn, *P* = 0.03; autumn *vs*. winter, *P* < 0.01). In addition, TBR was higher in summer or autumn than in spring (spring *vs*. summer, *P* = 0.02; spring *vs*. autumn, *P* < 0.01).

**Fig. 3:**
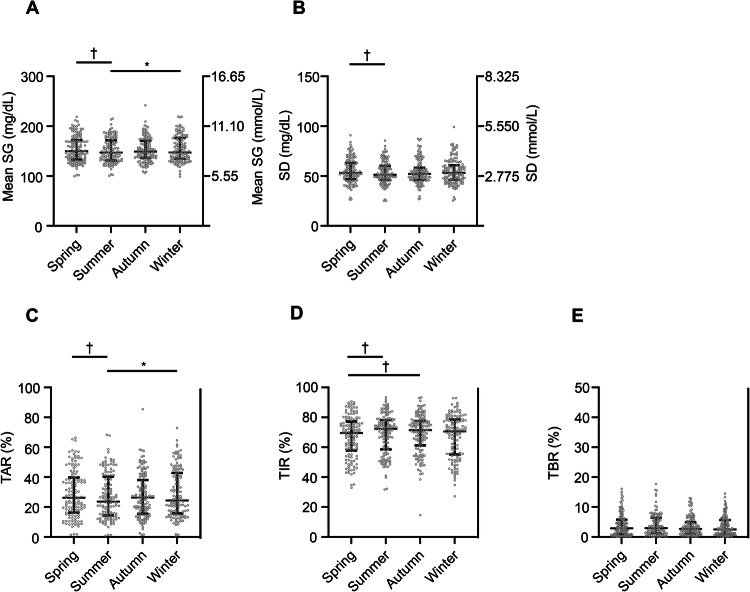
Seasonal fluctuations in CGM metrics for the SAP group of subjects. Seasonal fluctuations in mean SG (**A**), SD (**B**), TAR (**C**), TIR (**D**), and TBR (**E**) are shown. *P < 0.05 for comparisons with the value for winter; †*P* < 0.05 for comparisons with the value for spring (One-way repeated measures analysis of variance (ANOVA) with Bonferroni’s correction). *CGM* continuous glucose monitoring, *SAP* sensor augmented pump, *SG* sensor glucose, *SD* standard deviation, *TAR* time above range, *TIR* time in range, *TBR* time below range

In contrast, in the SAP group, seasonal fluctuations were not detected in TBR. Mean SG and TAR was lower in summer than in spring or winter, TIR was higher in summer or autumn than in spring, and SD was lower in summer than in spring (mean SG: spring *vs*. summer, *P* < 0.01; summer *vs*. winter, *P* = 0.02) (TAR: spring *vs*. summer, *P* = 0.01; summer *vs*. winter, *P* = 0.02) (TIR: spring *vs*. summer, *P* < 0.01; spring *vs*. autumn, *P* = 0.01) (SD: spring *vs*. summer, *P* < 0.01) (Fig. 3, Table 2).

## Discussion

We here found that individuals with T1DM showed seasonal fluctuations in CGM metrics, with higher mean SG, TAR, and SD and lower TIR values in spring or winter than in summer or autumn. As far as we are aware, no study has previously investigated such seasonal fluctuations.

Seasonal fluctuations in HbA_1c_ levels have been described for various geographic regions and found to be higher during cold seasons and lower during warm seasons [11–15]. Many Western countries experience distinct seasons, as does Japan, which has four typical seasons. The location in which this study was performed, Kobe, experiences a warm summer and autumn and a cold spring and winter (Supplementary Fig. 1). Physiological or metabolic factors related to ambient temperature, changes in diet or activity, and social conventions are thought to contribute to seasonal fluctuations in HbA_1c_ levels. We found that the seasonal fluctuations in CGM metrics such as mean SG, TAR, TIR, and SD were consistent with those in HbA_1c_ levels observed in both the present and previous studies. Moreover, it was suggested that mean SG, TAR, TIR, and SD were more responsive to changes of season than was HbA_1c_. In addition, seasonal fluctuations in CGM metrics were characterized by an increase in the hyperglycemic range and increased glycemic variability in spring or winter, regardless of CGM type.

SD, which is often adopted as a measure of glycemic variability, has previously been associated with an increased risk of diabetic complications and mortality [22–25]. A previous study also found a positive correlation between SD and TAR and a negative correlation between SD and TIR for individuals with T1DM [26]. In addition, TAR and TIR showed a much greater correlation with mean SG than with HbA_1c_ levels [27]. Our analysis revealed the presence of seasonal fluctuations in the SD of mean SG. Given that SD tends to be associated with other CGM metrics but not with HbA_1c_, the changes in SD during spring and winter may result in the corresponding increases in mean SG and TAR. A targeted reduction in glycemic variability during the cold seasons might therefore be expected to result in a lowering of TAR and improvement in glycemic management without an increase in the frequency of hypoglycemia.

Dietary intake and resting metabolic rate manifest seasonal changes, being higher in winter and lower in summer in Japan [28]. In regions with four distinct seasons, the resting metabolic rate increases in winter as an adaptation to maintain body temperature in the cold climate. In addition, the decline in the number of daylight hours in winter results in a decrease in outdoor activities [28]. Furthermore, mean outdoor temperature has been found to be associated with seasonal fluctuations in HbA_1c_ levels, with temperature-related physiological and metabolic factors having been proposed as the main determinants of such seasonal fluctuations, although individual lifestyle is also an important contributing factor [12, 29]. Our present demonstration of seasonal fluctuations in CGM metrics suggests that adjustment of insulin regimens should take into account seasonal changes in diet and activity levels in order to reduce glycemic variability.

We found that the isCGM group showed seasonal variation in TBR, which increased in summer and autumn compared with spring, whereas the frequency of hypoglycemia did not change similarly in the SAP group. The larger number of individuals in the isCGM group than in the SAP group may have influenced this difference. However, individuals using isCGM should pay particular attention to the potential development of hypoglycemia in summer and autumn.

Seasonal fluctuations in HbA_1c_ levels deviated somewhat from those in CGM metrics in the present study. HbA_1c_ is thought to reflect the average blood glucose level over the previous several months, whereas CGM metrics reflect the situation on the day of measurement. Indeed, TAR and TIR were previously shown to be more highly correlated with mean glucose levels than with HbA_1c_ levels [27]. CGM metrics may therefore be more sensitive than HbA_1c_ for detection of seasonal changes in glycemic control. Another possible explanation for the difference in the seasonal patterns of CGM metrics and HbA_1c_ is that several of the study subjects visited the hospital only every 3 months. Whereas CGM data were available for each month even for such individuals, HbA_1c_ data for subjects who visited every 3 months were excluded, possibly giving rise to the disparity between the seasonal fluctuations in CGM metrics and those in HbA_1c_ levels.

Although a multitude of factors, such as metabolism, extracurricular activities, and interactions among CGM metrics, may contribute to the findings of this study, further research is needed to validate these hypotheses.

Our study has several limitations. First, the study included only Japanese individuals, with individuals of other ethnicities or from other climatic regions thus not being considered. We were also able to examine the temperature trends only in Kobe, where our facility is located. Second, there were slight differences in the measurement methods and accuracy between isCGM and rtCGM in this study, with the mean absolute relative difference (MARD) of the FreeStyle Libre CGM device being 11.4% and that of the Enlite Sensor being 14.2% [30]. However, none of the study subjects changed CGM device during the study period, and this difference in MARD was considered to have little impact on the results. Third, we were not able to investigate changes in insulin dosage during the study because most of the subjects used carbohydrate counting and it was therefore difficult to obtain insulin dosage data for all individuals. Moreover, detailed information regarding the changes in lifestyle and BMI was not available. For individuals with T1DM who change their insulin dosage based on their lifestyle, it is possible that their insulin dosage may also fluctuate with the seasons, with additional studies being required to provide further insight into and to address this issue. Finally, based on the results of the post-hoc power analysis, it was determined that the sample size employed in this retrospective study possessed a relative power (76% with an alpha value of 0.05) to detect the difference in mean SG between spring and autumn, despite the absence of prior investigations into seasonal variations in CGM metrics. Nevertheless, it should be noted that the sample size and statistical power were not predetermined prior to the initiation of the study.

In routine practice, it is important to consider intraday and diurnal variations when utilizing CGM metrics. This research provides a novel approach to understanding and applying CGM data, while simultaneously accounting for seasonal fluctuations. These seasonal fluctuations in CGM metrics may be considered in future studies performed to evaluate the favorable impact of CGM on glycemic management in individuals with T1DM.

In conclusion, our study has demonstrated seasonal fluctuation of CGM metrics including mean SG, TAR, TIR, and SD in individuals with T1DM. The use of CGM metrics may be more sensitive than that of HbA_1c_ for detection of seasonal changes in glycemic control. Consideration of seasonal fluctuations in CGM metrics may therefore improve glycemic control and lower the risk of hypoglycemia, allowing the prevention of complication progression in routine clinical practice.

## Supplementary Information

Supply Fig.1

Supplementary Information

## References

[1] C. Fabris, L. Heinemann, R. Beck, C. Cobelli, B. Kovatchev, Estimation of hemoglobin A1c from continuous glucose monitoring data in individuals with type 1 diabetes: is time in range all we need? Diab. Technol. Ther. **22**, 501–508 (2020). 10.1089/dia.2020.0236. 10.1089/dia.2020.0236 PMC7336887 32459124

[2] R.I.G. Holt, J.H. DeVries, A. Hess-Fischl, I.B. Hirsch, M.S. Kirkman, T. Klupa et al. The management of type 1 diabetes in adults. a consensus report by the American Diabetes Association (ADA) and the European Association for the Study of Diabetes (EASD). Diab. Care **44**, 2589–2625 (2021). 10.2337/dci21-0043. 10.2337/dci21-0043 34593612

[3] A. Advani, Positioning time in range in diabetes management. Diabetologia **63**, 242–252 (2020). 10.1007/s00125-019-05027-0. 31701199 10.1007/s00125-019-05027-0

[4] M. Valenzano, I. Cibrario Bertolotti, A. Valenzano, G. Grassi, Time in range-A1c hemoglobin relationship in continuous glucose monitoring of type 1 diabetes: a real-world study. BMJ Open Diab. Res. Care [Internet] **9**, e001045 (2021). 10.1136/bmjdrc-2019-001045. 33514530 10.1136/bmjdrc-2019-001045 PMC7849891

[5] J. Lu, X. Ma, J. Zhou, L. Zhang, Y. Mo, L. Ying et al. Association of time in range, as assessed by continuous glucose monitoring, with diabetic retinopathy in type 2 diabetes. Diab. Care **41**, 2370–2376 (2018). 10.2337/dc18-1131. 10.2337/dc18-1131 30201847

[6] R.W. Beck, R.M. Bergenstal, T.D. Riddlesworth, C. Kollman, Z. Li, A.S. Brown et al. Validation of time in range as an outcome measure for diabetes clinical trials. Diab. Care **42**, 400–405 (2019). 10.2337/dc18-1444. 10.2337/dc18-1444 PMC6905478 30352896

[7] M.A. Rotondi, O. Wong, M. Riddell, B. Perkins, Population-level impact and cost-effectiveness of continuous glucose monitoring and intermittently scanned continuous glucose monitoring technologies for adults with type 1 diabetes in Canada: a modeling study. Diab. Care **45**, 2012–2019 (2022). 10.2337/dc21-2341. 10.2337/dc21-2341 PMC9472499 35834175

[8] J. Šoupal, L. Petruželková, G. Grunberger, A. Hásková, M. Flekač, M. Matoulek et al. Glycemic outcomes in adults with T1D are impacted more by continuous glucose monitoring than by insulin delivery method: 3 years of follow-up from the COMISAIR study. Diab. Care **43**, 37–43 (2020). 10.2337/dc19-0888. 10.2337/dc19-0888 31530663

[9] N.A. ElSayed, G. Aleppo, V.R. Aroda, R.R. Bannuru, F.M. Brown, D. Bruemmer et al. 7. Diabetes technology: standards of care in diabetes-2023. Diab. Care **46**, S111–S127 (2023). 10.2337/dc23-S007. 10.2337/dc23-S007 PMC9810474 36507635

[10] M.I. Maiorino, S. Signoriello, A. Maio, P. Chiodini, G. Bellastella, L. Scappaticcio et al. Effects of continuous glucose monitoring on metrics of glycemic control in diabetes: a systematic review with meta-analysis of randomized controlled trials. Diab. Care **43**, 1146–1156 (2020). 10.2337/dc19-1459. 10.2337/dc19-1459 32312858

[11] A. Gikas, A. Sotiropoulos, V. Pastromas, A. Papazafiropoulou, O. Apostolou, S. Pappas, Seasonal variation in fasting glucose and HbA1c in patients with type 2 diabetes. Prim. Care Diab. **3**, 111–114 (2009). 10.1016/j.pcd.2009.05.004. 10.1016/j.pcd.2009.05.004 19535310

[12] H. Sakura, Y. Tanaka, Y. Iwamoto, Seasonal fluctuations of glycated hemoglobin levels in Japanese diabetic patients. Diab. Res Clin. Pr. **88**, 65–70 (2010). 10.1016/j.diabres.2009.12.011. 10.1016/j.diabres.2009.12.011 20047769

[13] Y. Zhang, M. Tong, B. Wang, Z. Shi, P. Wang, L. Li et al. Geographic, gender, and seasonal variation of diabetes: a nationwide study with 1.4 million participants. J. Clin. Endocrinol. Metab. **106**, e4981–e4992 (2021). 10.1210/clinem/dgab543. 34314489 10.1210/clinem/dgab543

[14] T. Higgins, S. Saw, K. Sikaris, C.L. Wiley, G.C. Cembrowski, A.W. Lyon et al. Seasonal variation in hemoglobin A1c: is it the same in both hemispheres? J. Diab. Sci. Technol. **3**, 668–671 (2009). 10.1177/193229680900300408. 10.1177/193229680900300408 PMC2769947 20144310

[15] H. Ishii, H. Suzuki, T. Baba, K. Nakamura, T. Watanabe, Seasonal variation of glycemic control in type 2 diabetic patients. Diab. Care **24**, 1503 (2001). 10.2337/diacare.24.8.1503. 10.2337/diacare.24.8.1503 11473100

[16] R. Bouchi, T. Sugiyama, A. Goto, M. Ohsugi, N. Yoshioka, H. Katagiri et al. Impact of COVID-19 pandemic on behavioral changes and glycemic control and a survey of telemedicine in patients with diabetes: a multicenter retrospective observational study. J. Diab. Investig. **14**, 994–1004 (2023). 10.1111/jdi.14027. 10.1111/jdi.14027 PMC10360386 37183588

[17] Y. Hosomi, C. Munekawa, Y. Hashimoto, T. Okamura, F. Takahashi, R. Kawano et al. The effect of COVID-19 pandemic on the lifestyle and glycemic control in patients with type 1 diabetes: a retrospective cohort study. Diabetol. Int. **13**, 85–90 (2022). 10.1007/s13340-021-00507-4. 33898153 10.1007/s13340-021-00507-4 PMC8054854

[18] Japan Meteorological Agency. [cited 2024 Apr 18]. Available from: https://www.data.jma.go.jp/obd/stats/etr.n/index.php.

[19] L. Blonde, G.E. Umpierrez, S.S. Reddy, J.B. McGill, S.L. Berga, M. Bush, et al. American Association of Clinical Endocrinology Clinical Practice Guideline: developing a diabetes mellitus comprehensive care plan-2022 update. Endocr. Pr. **28**, 923–1049 (2022). 10.1016/j.eprac.2022.08.002. 10.1016/j.eprac.2022.08.002 PMC10200071 35963508

[20] N.A. ElSayed, G. Aleppo, V.R. Aroda, R.R. Bannuru, F.M. Brown, D. Bruemmer et al. 11. Chronic kidney disease and risk management: standards of care in diabetes-2023. Diab. Care **46**, S191–S202 (2023). 10.2337/dc23-S011. 10.2337/dc23-S011 PMC9810467 36507634

[21] Y. Kanda, Investigation of the freely available easy-to-use software “EZR” for medical statistics. Bone Marrow Transpl. **48**, 452–458 (2013). 10.1038/bmt.2012.244. 10.1038/bmt.2012.244 PMC3590441 23208313

[22] S. Lee, J. Zhou, W.T. Wong, T. Liu, W.K.K. Wu, I.C.K. Wong et al. Glycemic and lipid variability for predicting complications and mortality in diabetes mellitus using machine learning. BMC Endocr. Disord. **21**, 94 (2021). 10.1186/s12902-021-00751-4. 33947391 10.1186/s12902-021-00751-4 PMC8097996

[23] S. Lee, T. Liu, J. Zhou, Q. Zhang, W.T. Wong, G. Tse, Predictions of diabetes complications and mortality using hba1c variability: a 10-year observational cohort study. Acta Diabetol. **58**, 171–180 (2021). 10.1007/s00592-020-01605-6. 32939583 10.1007/s00592-020-01605-6

[24] E.S. Scott, R.T. McGrath, A.S. Januszewski, D. Calandro, A.A. Hardikar, D.N. O’Neal et al. HbA1c variability in adults with type 1 diabetes on continuous subcutaneous insulin infusion (CSII) therapy compared to multiple daily injection (MDI) treatment. BMJ Open **9**, e033059 (2019). 10.1136/bmjopen-2019-033059. 31888933 10.1136/bmjopen-2019-033059 PMC6937034

[25] Y. Mo, J. Lu, J. Zhou, Glycemic variability: measurement, target, impact on complications of diabetes and does it really matter? J. Diab. Investig. **15**, 5–14 (2024). 10.1111/jdi.14112. 10.1111/jdi.14112 PMC10759720 37988220

[26] M. Ohigashi, K. Osugi, Y. Kusunoki, K. Washio, S. Matsutani, T. Tsunoda et al. Association of time in range with hemoglobin A1c, glycated albumin and 1,5-anhydro-d-glucitol. J. Diab. Investig. **12**, 940–949 (2021). 10.1111/jdi.13437. 10.1111/jdi.13437 PMC8169363 33058513

[27] D. Rodbard, Continuous glucose monitoring metrics (Mean Glucose, time above range and time in range) are superior to glycated haemoglobin for assessment of therapeutic efficacy. Diab. Obes. Metab. **25**, 596–601 (2023). 10.1111/dom.14906. 10.1111/dom.14906 36314133

[28] N. Tanaka, T. Okuda, H. Shinohara, R.S. Yamasaki, N. Hirano, J. Kang et al. Relationship between seasonal changes in food intake and energy metabolism, physical activity, and body composition in young Japanese women. Nutrients **14**, 506 (2022). 10.3390/nu14030506. 35276865 10.3390/nu14030506 PMC8838489

[29] E. Yoshimura, E. Tajiri, Y. Hatamoto, S. Tanaka, Changes in season affect body weight, physical activity, food intake, and sleep in female college students: a preliminary study. Int J. Environ. Res Public Health **17**, 8713 (2020). 10.3390/ijerph17238713. 33255205 10.3390/ijerph17238713 PMC7727682

[30] D. Rodbard, Continuous glucose monitoring: a review of successes, challenges, and opportunities. Diab. Technol. Ther. **18**, S3–S13 (2016). 10.1089/dia.2015.0417. 10.1089/dia.2015.0417 PMC4717493 26784127

