# Supplementary figures and images for: Seasonal fluctuations of CGM metrics in individuals with type 1 diabetes using an intermittently scanned CGM device or sensor augmented pump
Source: Endocrine. 2024 Jul 25;87(1):85–93. doi: 10.1007/s12020-024-03971-5 (PMC11739268; doi:10.1007/s12020-024-03971-5)

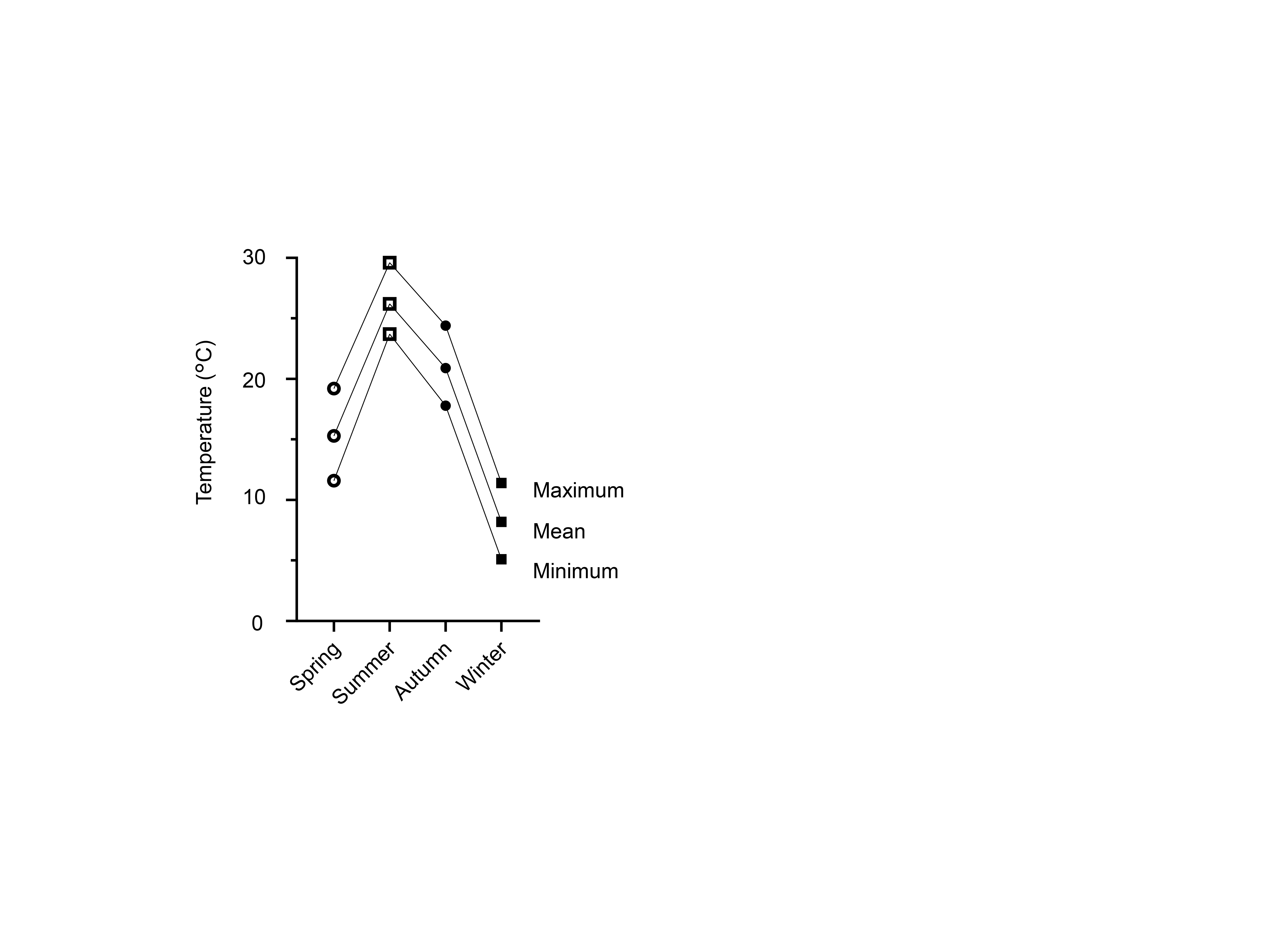

Supplement: Supplementary file 1 — Supply Fig.1 [file 12020_2024_3971_MOESM1_ESM.tif]
